# Addressing influenza’s underestimated burden – Iberian experts call to action

**DOI:** 10.1186/s12879-023-08277-x

**Published:** 2023-05-08

**Authors:** Ángel Gil-de-Miguel, Javier Díez-Domingo, Federico Martinón-Torres, Esther Redondo Margüello, Raúl Ortiz de Lejarazu Leonardo, Tomàs Pumarola, Jaime Correia de Sousa, Carlos Rabaçal, João Raposo, Carlos Robalo Cordeiro, Filipe Froes

**Affiliations:** 1 Public Health and Medical Specialties Department, Health Sciences Faculty, Rey Juan Carlos University, Madrid, Spain; 2 Vaccine Research Department, University of Valencia, Valencia, Spain; 3 Translational Pediatrics and Infectious Diseases, Hospital Clínico Universitario and Universidad de Santiago de Compostela, Galicia, Spain; 4 Genetics, Vaccines and Pediatric Infectious Diseases Research Group (GENVIP), Instituto de Investigación Sanitaria de Santiago and Universidad de Santiago de Compostela (USC), Galicia, Spain; 5 CIBER Enfermedades Respiratorias, Instituto de Salud Carlos III, Madrid, Spain; 6 International Health Center Madrid Health, City Council of Madrid, Madrid, Spain; 7 National Influenza Centre, Hospital Clínico Universitario, Universidad de Valladolid, Valladolid, Spain; 8 Department of Microbiology, Hospital Universitari Vall d’Hebron, Barcelona, Spain; 9 Life and Health Sciences Research Institute (ICVS), School of Medicine, PT Government Associate Laboratory, University of Minho, Braga Portugal. ICVS/3B’s, Braga/Guimarães, Portugal; 10 Vila Franca de Xira Hospital, Lisbon, Portugal; 11 APDP and Nova Medical School, Lisbon, Portugal; 12 Pulmonology Department, Coimbra University Hospital, University of Coimbra, Coimbra, Portugal; 13 Thorax Department, ICU, Centro Hospitalar Universitário Lisboa Norte, Lisbon, Portugal; 14 Universidad Rey Juan Carlos, Avda. de Atenas, s/n, 28922 Alcorcón, Madrid, Spain

**Keywords:** Influenza, Burden, Epidemiology, Spain, Portugal, Hospitalization, Vaccination, Prevention, Surveillance, Retrospective studies

## Abstract

Having a proper understanding of the impact of influenza is a fundamental step towards improved preventive action. This paper reviews findings from the Burden of Acute Respiratory Infections study on the burden of influenza in Iberia, and its potential underestimation, and proposes specific measures to lessen influenza’s impact.

## Introduction

Influenza is a viral infection which may trigger severe complications, potentially leading to hospitalization or death [1–3]. Complications go beyond the respiratory illness and can include exacerbation of underlying chronic conditions, functional decline, vulnerability to secondary bacterial infections, as well as rare complications affecting other organ systems, such as the nervous and cardiovascular systems [3]. The importance of preventing influenza is recognized by the scientific community and reflected in the World Health Organization (WHO) recommendation of annual vaccination for health-care workers (HCW) and at-risk individuals, such as children aged between 6 and 59 months, pregnant women, elderly persons, individuals aged > 6 months with certain chronic medical conditions, and residents of nursing homes and the disabled [4, 5]. In the United States of America (USA), the Centers for Disease Control and Prevention (CDC) further recommends it for all people aged 6 months and older [6]. Since 2021, the Spanish Paediatric Association recommends universal vaccination to children aged between 6 and 59 months [7, 8], and states it would also be advisable to vaccinate older children, with or without chronic medical conditions [8]. Still, not all European Countries cover these groups in their vaccination programmes and vaccination coverage rates (VCR) are regularly below the WHO target [9, 10]. In addition, the burden of influenza tends to be underestimated due to the poor influenza diagnosis and coding practices or limited perspective of the analyses, focusing only on the hospital setting, [11, 12] and often lacks granularity on the patient’s age and comorbidities [13].

This paper aims at reviewing the evidence of the burden of influenza in Iberia, and its potential underestimation factor, obtained from the Burden of Acute Respiratory Infections (BARI) study and proposing specific measures to lessen influenza’s impact, endorsed by a group of eleven recognized experts in Spain and Portugal.

## What was the BARI study?

The BARI study assessed the clinical and economic burden of influenza in Spain and Portugal. National Health Service (NHS) hospitalizations coded with influenza-specific diagnosis were compared with estimates on the excess hospitalization and mortality attributable to influenza based on time series ecological models. Results have been published for Spain and Portugal [14, 15]. Complementarily, an electronic medical records (EMR) database from four Spanish regions during one season (2017/18) was analysed, shedding light on the direct healthcare burden of medically attended influenza, beyond hospitalization and using longitudinal health care data [16].

## What did we learn from the BARI study?

### Severe influenza still creates a substantial pressure on NHS resources

Influenza was estimated to be responsible for 34,894 respiratory and/or cardiovascular (C&R) hospitalizations on average over 9 influenza seasons in public hospitals per year in Spain and 5,356 in Portugal [14, 15]. In the deadliest season (2014/15), 24,268 excess all-cause deaths attributable to influenza were estimated in Spain and 5,016 in Portugal [14, 15]. It led to a substantial pressure on NHS resources due to influenza during winter. Patients stayed at the hospital on average 9.4 days in Spain and 10.7 days in Portugal, both above the respective national means for all-causes and ages [14, 15].

### Comorbidities are an important risk factor, regardless of age

Patients with comorbidities accounted for 59.0%/65.6% of NHS hospitalizations coded as influenza and for 65.7%/78.6% of their costs (in Spain/Portugal).^14,15^ Although most hospitalizations and deaths were driven by population aged ≥ 65 years old, population from 50 to 64 years with comorbidities was a particularly vulnerable group [14, 15]. In both countries, the VCR in this age group is lower than in the ≥ 65 years old group, namely, in season 2017/2018: 55.9% in those ≥ 65 years vs. 22.1% in population aged 60–64 years in Spain [17]; and 61.2% vs. 31.8% in Portugal [18], respectively. Hospitalized patients with comorbidities had increased lengths-of-stay, use of mechanical ventilation, and in-hospital mortality rates, compared to hospitalized patients without comorbidities, even after controlling for age. The role of comorbidities is particularly worthy of attention and action. The prevalence of comorbidities and of multimorbidity is increasing over time in many European countries, increasing the portion of population at high-risk for influenza’s severe complications.

### The impact of influenza goes beyond respiratory complications and is widely underestimated

Our findings further support that the burden of influenza goes beyond respiratory complications, [3] and that its impact is not adequately captured by the current hospitalization and deaths codification practices, nor by analysing only other respiratory diagnoses. Unlike what was observed during the COVID-19 pandemic – where testing became more generalized –data from the BARI study suggests that many hospitalizations and deaths triggered by influenza were still left unidentified [14, 15]. Hospitalizations coded as influenza represented only 37.4%/27.3% of the estimated excess C&R hospitalizations attributable to influenza in all-age group and 26.3%/14.0% in population aged ≥ 65 (in Spain/Portugal). Deaths observed during the NHS hospitalizations coded as influenza represented only 5.3%/3.4% and 4.3%/2.5% of the estimated excess all-cause deaths attributable to influenza, in all age groups and in population aged ≥ 65, respectively (in Spain/Portugal).^14,15^

### Influenza still generates a high economic burden

We estimated a mean annual cost of excess NHS hospitalizations that could be attributed to influenza of €142.9 million (min: 68.6; max: 216.6) in Spain and €15.2 million (min: 1.3; max: 24.9) in Portugal. Population aged ≥ 65 years accounted for three-fourths of the cost, and younger adults (19 to 64 years) accounted for one-fifth [14, 15]. Estimates are conservative as they consider only NHS hospitalizations. The analysis of EMR from four Spanish regions has found evidence that population aged between 18 and 64 years old generated the highest share of costs to the NHS (61.9%) when all healthcare cost were considered, and not only hospitalization [16]. Individuals with comorbidities accounted for most of the costs (67.1%), regardless of age [16].

## What are our proposed actions?

### Rethink respiratory infectious diseases surveillance systems considering learnings from the pandemics

Evidence is solid on the need for more testing and improved coding of influenza cases, as was performed during the COVID-19 pandemic. Surveillance systems for influenza – and overall for respiratory infectious diseases – should be reassessed considering the learnings from the pandemics [19]. Amongst other factors, we propose that:

1. Coding teams incorporate professionals with experience in managing respiratory disease and include proper training to address the barriers in codifying such diseases.
2. When feasible, preference is given to multiplex testing to enable simultaneous detection and differentiation between respiratory viruses.
3. The testing capacities are increased, through time, particularly at primary care level. Despite the observed improvements through strict diagnosis protocol to use influenza code, testing can enable a better detection of influenza.
4. Surveillance and burden of influenza data is stratified both by age and comorbidities status, through timely and publicly available reports, to enable more informed decision making.
5. Learnings from pandemic years are incorporated in the surveillance systems in place. Countries should transition to integrated national sentinel respiratory surveillance systems, including all respiratory viruses; increase their representativeness; and ensure all-year systematic sampling integrating data from primary care, hospitals, microbiology, and genomic laboratories.
6. Historical multipliers are used when communicating the burden of influenza, as performed in the USA, [20] to mitigate the impact on public perception of the real risk of influenza due to its under estimation. Specific mathematical models would have to be developed for each country.

### Improve awareness on influenza complications and on the importance of its prevention

In order to ensure the necessary preventive actions are taken by all vested parties, it is necessary to properly communicate the potential influenza complications and the importance of prevention through vaccination. We propose that:

1. More focus is given to generating awareness of HCW as possible agents of transmission to increase their VCR.
2. There is an increase in the adoption of multidisciplinary approaches by HCW (physicians, nurses, pharmacists) to reduce influenza’s burden also in population aged < 65 years with comorbidities, as it should correspond to the active population that visits health centres and pharmacies.
3. More communication is done at primary care level, namely with up-to-date information on the complications that influenza can trigger in each risk-group. Striking evidence, such that the incidence of hospital admissions for acute myocardial infarction were found to be at least six times higher during the 7 days after laboratory confirmation of influenza infection [21, 22], must be acknowledged by clinicians and patients. Increasing awareness amongst HCW is also a mean to achieve higher VCR across the population.
4. Campaigns are conducted to raise awareness of the general population on the importance of influenza vaccination. When possible, targeted messages tailored to the different population segments are recommended to achieve maximum impact, considering factors such as the risk profile, potential vaccine hesitancy, amongst others.

### Further invest on vaccination as a high value public health tool

Seasonal influenza vaccination has been demonstrated to be a valuable public health preventive measure, being safe, effective, and cost-effective [23]. There is evidence that, from a societal perspective, vaccination against seasonal influenza is cost-effective and reduces costs for the health systems, not only for the elderly, but also for other groups such as children, pregnant and postpartum women, high risk groups, and, in some cases, healthy working age adults and HCW [23–27]. This is well known and reflected in vaccination guidances [4, 23].

But VCR need to reach the established targets and vaccines need to be selected based on demonstrated efficacy in preventing infection as well as in preventing complications attributable to influenza, such as cardiorespiratory hospitalizations or all cause hospitalizations, which may differ according to patient’s characteristics, and circulating strains [23]. For example, studies for the elderly population report that influenza vaccination is cost-effective regardless of the type of vaccine, but the magnitude of cost-effectiveness is higher in high-dose TIV and QIV than in standard-dose TIV [28, 29]. We must ensure that the right vaccines are administered to the right at-risk population to achieve better protection, enabling higher cost savings to the NHS and lower health care resource utilization.

Overall, we defend that the 75% VCR target should be reached in all at-risk population. We identify the following groups as critical to prioritize:

1. Population aged 65 years old or above (keep or improve vaccination to be within or above WHO target).
2. People with comorbidities, irrespective of age.
3. HCW’s.
4. Pregnant women.
5. Children aged < 5 years old.

## Conclusions

Influenza’s burden is underestimated by the reported data, leading to an insufficient recognition of its impact on our societies. Nonetheless, the impact is there. Our study shows the need to lessen influenza’s impact in Spain and Portugal, particularly in the elderly and in people with comorbidities, regardless of age. We hereby urge decision-makers to rethink surveillance systems, considering learnings from pandemics, increase awareness and quickly achieve the target VCR, ensuring that the right vaccines are given to the right people.

## Data Availability

The data that support the findings of the BARI study are available from IQVIA, but restrictions apply to the availability of these data, which were used under license for the current study, and so are not publicly available. Data are however available from the authors upon reasonable request and with permission of IQVIA. Those wishing to request the data from this study should contact Mafalda Carmo (mafalda.carmo@iqvia.com).

## References

[1] Troeger CE, Blacker BF, Khalil IA (2019). Mortality, morbidity, and hospitalisations due to influenza lower respiratory tract infections, 2017: an analysis for the global burden of Disease Study 2017. The Lancet Respiratory Medicine.

[2] Paules C, Subbarao K, Influenza (2017). The Lancet.

[3] Macias AE, McElhaney JE, Chaves SS (2021). The disease burden of influenza beyond respiratory illness. Vaccine.

[4] WHO. World Health Organization. https://www.euro.who.int/en/health-topics/communicable-diseases/influenza/vaccination. Accessed July 2021, 2021.

[5] WHO. World Health Organization (2022). Vaccines against influenza: WHO position paper, May 2022. Wkly Epidemiol Rec.

[6] CDC, Vaccine Effectiveness. How Well Do Flu Vaccines Work? https://www.cdc.gov/flu/vaccines-work/vaccineeffect.htm. Published 2021. Updated Page last reviewed: September 17, 2021. Accessed Page last accessed: October 17, 2021.

[7] CAV-AEP. Vacunación frente a la gripe estacional en la infancia. Recomendaciones del CAV-AEP 2021–2022. In: Vacunas, CAd, editors. 20 de septiembre de 2021. Asociación Española de Pediatría; 2021.

[8] CAV-AEP. Recomendaciones del CAV-AEP para la campaña antigripal 2022–2023 in: Vacunas CAd. ed: Asociación Española de Pediatría; 2022.

[9] Jorgensen P, Mereckiene J, Cotter S, Johansen K, Tsolova S, Brown C (2018). How close are countries of the WHO European Region to achieving the goal of vaccinating 75% of key risk groups against influenza? Results from national surveys on seasonal influenza vaccination programmes, 2008/2009 to 2014/2015. Vaccine.

[10] WHO. European Health Information Gateway. In: Organization WH, ed.

[11] Hamilton MA, Calzavara A, Emerson SD (2021). Validating International classification of Disease 10th revision algorithms for identifying influenza and respiratory syncytial virus hospitalizations. PLoS ONE.

[12] Thompson WW, Moore MR, Weintraub E (2009). Estimating Influenza-Associated deaths in the United States. Am J Public Health.

[13] de Courville C, Cadarette SM, Wissinger E, Alvarez FP (2022). The economic burden of influenza among adults aged 18 to 64: a systematic literature review. Influenza Other Respir Viruses.

[14] Froes F, Carmo M, Lopes H (2022). Excess hospitalizations and mortality associated with seasonal influenza in Portugal, 2008–2018. BMC Infect Dis.

[15] Pumarola T, Díez-Domingo J, Martinón-Torres F (2023). Excess hospitalizations and mortality associated with seasonal influenza in Spain, 2008–2018. BMC Infect Dis.

[16] Gil-de-Miguel Á, Martinón-Torres F, Díez-Domingo J (2022). Clinical and economic burden of physician-diagnosed influenza in adults during the 2017/2018 epidemic season in Spain. BMC Public Health.

[17] Coberturas de vacunación de gripe. https://pestadistico.inteligenciadegestion.sanidad.gob.es/publicoSNS/I/sivamin/sivamin. Accessed Consulted on 20th December 2022.

[18] Vacinómetro®. *Resultados da 9.ª Edição do Vacinómetro® época gripal 2017/2018* Lisboa, 29 de janeiro de 2018: Sociedade Portuguesa de Pneumologia (SPP) e Associação Portuguesa de Medicina Geral e Familiar (APMGF);2018.

[19] Rigoine de Fougerolles T, Puig-Barbera J, Kassianos G (2022). A comparison of coronavirus disease 2019 and seasonal influenza surveillance in five european countries: France, Germany, Italy, Spain and the United Kingdom. Influenza Other Respir Viruses.

[20] CDC, How. CDC Estimates the Burden of Seasonal Influenza in the U.S. https://www.cdc.gov/flu/about/burden/how-cdc-estimates.htm. Published 2019. Updated Page last reviewed: November 22, 2019. Accessed Page last accessed: May 02, 2022.

[21] Kwong JC, Schwartz KL, Campitelli MA (2018). Acute myocardial infarction after laboratory-confirmed influenza infection. N Engl J Med.

[22] Ohland J, Warren-Gash C, Blackburn R (2020). Acute myocardial infarctions and stroke triggered by laboratory-confirmed respiratory infections in Denmark, 2010 to 2016. Eurosurveillance.

[23] WHO. Seasonal influenza vaccines: an overview for decision-makers. In: Organization WH, ed. Geneva 2020.

[24] Peasah SK, Azziz-Baumgartner E, Breese J, Meltzer MI, Widdowson MA (2013). Influenza cost and cost-effectiveness studies globally–a review. Vaccine.

[25] Ting EEK, Sander B, Ungar WJ (2017). Systematic review of the cost-effectiveness of influenza immunization programs. Vaccine.

[26] Shields GE, Elvidge J, Davies LM (2017). A systematic review of economic evaluations of seasonal influenza vaccination for the elderly population in the European Union. BMJ Open.

[27] de Lejarazu Leonardo RO, Moraga-Llop F. Carga de gripe en la población pediátrica en España y los beneficios de la vacunación. Vacunas. 2023 Feb 18.

[28] Dilokthornsakul P, Lan LM, Thakkinstian A, Hutubessy R, Lambach P, Chaiyakunapruk N (2022). Economic evaluation of seasonal influenza vaccination in elderly and health workers: a systematic review and meta-analysis. eClinicalMedicine.

[29] Redondo E, Drago G, López-Belmonte JL (2021). Cost-utility analysis of influenza vaccination in a population aged 65 years or older in Spain with a high-dose vaccine versus an adjuvanted vaccine. Vaccine.

